# Metaphor in Sign Languages

**DOI:** 10.3389/fpsyg.2018.01025

**Published:** 2018-06-26

**Authors:** Irit Meir, Ariel Cohen

**Affiliations:** ^1^Department of Hebrew Language, University of Haifa, Haifa, Israel; ^2^Department of Communication Sciences and Disorders, University of Haifa, Haifa, Israel; ^3^Department of Foreign Literatures and Linguistics, Ben-Gurion University of the Negev, Beersheba, Israel

**Keywords:** metaphor, simile, iconicity, inhibition, Double Mapping Constraint, autonomous and dependent elements

## Abstract

Metaphor abounds in both sign and spoken languages. However, in sign languages, languages in the visual-manual modality, metaphors work a bit differently than they do in spoken languages. In this paper we explore some of the ways in which metaphors in sign languages differ from metaphors in spoken languages. We address three differences: (a) Some metaphors are very common in spoken languages yet are infelicitous in sign languages; (b) Body-part terms are possible in very specific types of metaphors in sign languages, but are not so restricted in spoken languages; (c) Similes in some sign languages are dispreferred in predicative positions in which metaphors are fine, in contrast to spoken languages where both can appear in these environments. We argue that these differences can be explained by two seemingly unrelated principles: the Double Mapping Constraint (Meir, 2010), which accounts for the interaction between metaphor and iconicity in languages, and Croft’s (2003) constraint regarding the autonomy and dependency of elements in metaphorical constructions. We further argue that the study of metaphor in the signed modality offers novel insights concerning the nature of metaphor in general, and the role of figurative speech in language.

## Introduction

Metaphor, the use of an item from one semantic domain in a different semantic domain in order to characterize the latter in terms of the former, is pervasive in human language and thought. Though it is often regarded as a poetic device used in figurative language to create special poetic effects, works on metaphor in the past several decades have demonstrated that metaphors are used in everyday use of language, and not only in language but in thought and action as well (Lakoff and Johnson, 1980). In fact, we cannot avoid using metaphors; all we need is to look and we will catch metaphors in many everyday utterances (note that *look* and *catch* are used metaphorically here). Since our potential experiences are infinite, yet the lexicon of any language is finite, the use of metaphor is a powerful way to refer to new situations by using the existing linguistic means that we have (e.g., *surfing* the inter*net*, a computer *mouse*, a space*ship*).

Furthermore, metaphor is not restricted to language; it is used in other domains of human cognition as well, such as mathematics (Nunez, 2008), visual art (Kennedy, 1982, 2008; Forceville, 2008), graphics, and music (Zbikowski, 2008).

Natural languages come in two modalities—spoken and signed. Both types of languages develop naturally in human communities, shaped by the special characteristics of the human brain and human capacity for language, by human cognition and by the communicative needs and constraints of human communities. The languages produced in the two modalities have many important properties in common, in their linguistic structures, processes, constraints and communicative functions (Sandler and Lillo-Martin, 2006). Since metaphor seems to be such a basic and pervasive cognitive process, we would expect to find it in both types of languages.

Yet the two types of languages differ markedly in their physical characteristics, and these physical characteristics entail some important linguistic differences between the two modalities. For example, in sign languages, both the articulations of the hands and their relation to space are directly perceivable, unlike those of the vocal tract, whose articulations are perceivable only indirectly, via the acoustic patterns created by air passing through different vocal tract configurations. Sign languages also fully exploit the existence of the two hands – phonologically, lexically, and at higher levels of structure. These two identical articulators can behave independently, and have no parallel in speech (see e.g., Sandler, 1993, 2006, 2013; Liddell, 2003; Crasborn, 2012).

These modality differences result in structural differences as well (see e.g., Meier et al., 2002). For example, sign languages exhibit more simultaneous structure on all linguistic levels (Sandler and Lillo-Martin, 2006; Vermeerbergen et al., 2007), while spoken languages show a tendency toward sequential structures. In addition, iconicity is more pervasive on all linguistic levels in sign languages than in spoken languages (Johnston and Schembri, 1999; Aronoff et al., 2005; Meir, 2010; Lepic et al., 2016).

What about metaphor? Would we expect languages in the two modalities to behave differently with respect to metaphorical expressions? Why should we, or why shouldn’t we, expect such differences? As we pointed out above, metaphors can be found in visual forms of communication and art. Therefore, we would expect to find them in visual languages too. However, metaphors in visual systems may work differently than metaphors in spoken languages. Kennedy (2008, p. 455) points out that a metaphor such as *a burning passion* works well in spoken language, but a picture of a burning person misses the point entirely. He points out that additional physical details, that cannot be avoided in a picture, are often extraneous and distracting. Kennedy further notices (ibid., p. 458) that in pictures one cannot distinguish between a metaphor (*my daughter is an angel*) and a simile (*my daughter is like an angel*)^1^.

Sign languages are both visual systems and linguistic systems. We might expect metaphor to work in a similar way in languages in general, building on the properties shared by all human languages. Yet if modality does play a role in shaping metaphors, as suggested above, then metaphors may work differently in the two types of languages.

Research on metaphors in sign languages reveals that metaphor is abundant in these languages. The seminal work of Wilcox (2000) and Taub (2001) on metaphor in sign languages showed that metaphorical mapping plays a central role in creating signs, especially signs for abstract concepts. Moreover, they show that the types of mappings found in ASL are those mentioned by Lakoff and Johnson (1980) as forming the basis for conceptual metaphors in spoken languages, such as: GOOD IS UP, THE FUTURE IS AHEAD, INTIMACY IS PROXIMITY, COMMUNICATION IS SENDING, UNDERSTANDING IS GRASPING, and many more. More recently, Roush (2016) examined eleven sub-mappings of location event-structure metaphors, which are claimed to be universal in spoken languages, and found that all of them are exhibited in signs from the ASL lexicon. These studies provide strong support for prevalence of conceptual metaphor in human language, regardless of modality.

Important insights can be obtained from the study of metaphorical gestures (e.g., Cienki and Müller, 2008), although gestures, unlike signs, usually co-occur with speech. While McNeill (1992) distinguishes between iconic and metaphorical gestures, Cienki and Müller (2008) argue that metaphorical gestures are in fact iconic. The iconic nature of gestures is particularly important, as it “affords different potentials than aural/oral expression does” (Müller and Cienki, 2009, p. 322).

**FIGURE 1:**
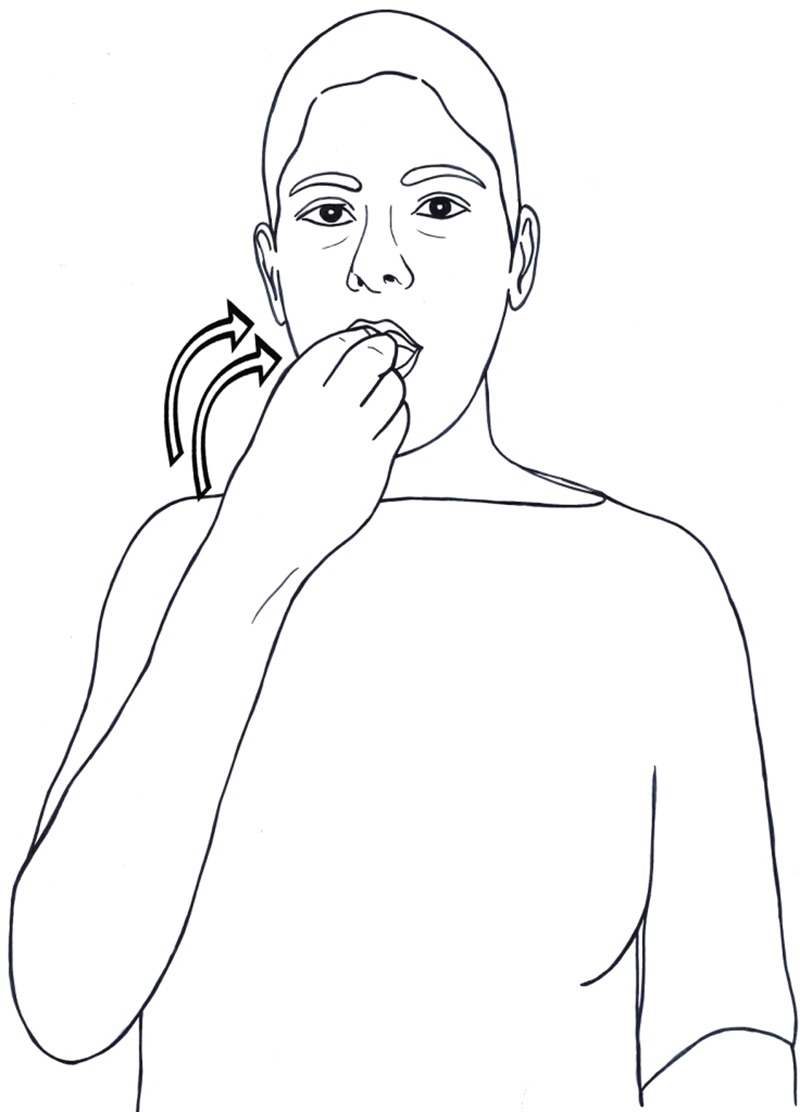
An iconic sign: EAT (ISL).

**FIGURE 2:**
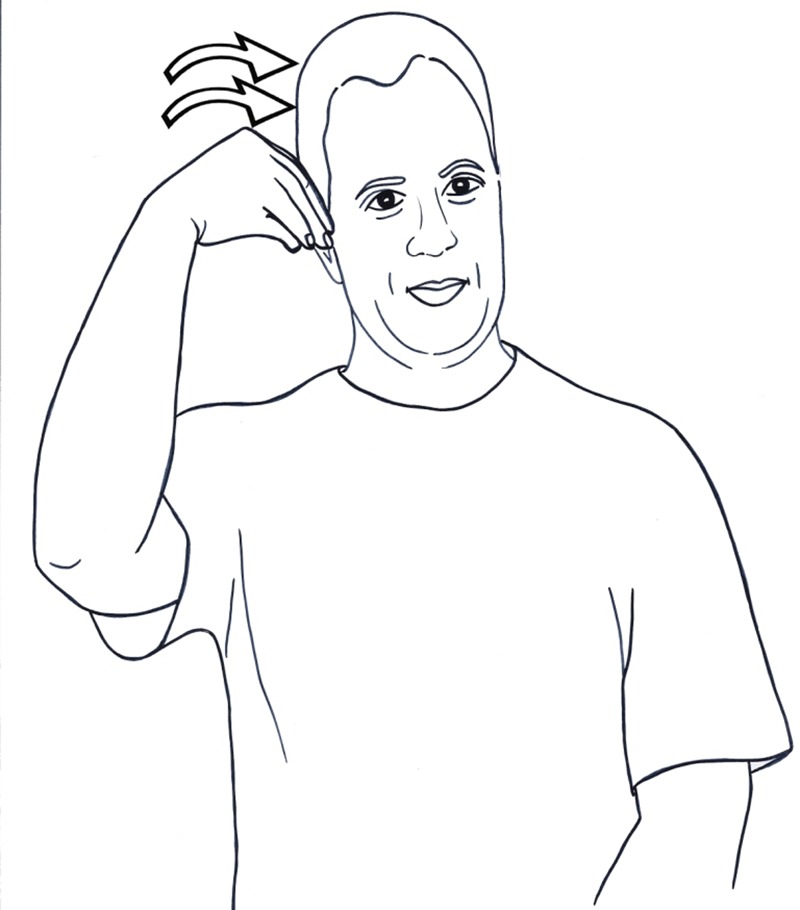
An iconic sign used metaphorically to represent an abstract action: LEARN (ISL).

Wilcox (2000) and Taub (2001) focus on the interaction of metaphor and iconicity in the structure of signs. They show how the different phonological components of a sign – its hand configuration, location and movement – can represent iconically some of the meaning components of that sign, and then can be metaphorically mapped to an abstract concept in a different semantic domain. For example, the sign EAT in Israeli Sign Language (ISL) has the form of a 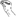 handshape, moving in a repeated movement toward the signer’s mouth. The form of the sign iconically represents holding a small object (by the 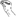 handshape), and putting it into the agent’s mouth (**Figure 1**). When the hand performs the same movement toward the temple, the sign means LEARN, represented iconically as the action of putting something inside one’s head (**Figure 2**). In this sign, the iconic representation is mapped onto the abstract domain of mental activities, which is characterized by putting objects (ideas, information, pieces of knowledge) into a container (the head).

Taub (2001) elaborated on the relationship between iconicity and metaphor, and suggested an explicit model capturing the relationship between the two. Specifically, she suggests that the creation of an iconic sign is a process of mapping elements of form to elements of meaning. And the creation of an iconic-metaphorical sign is shaped by double mapping: an iconic mapping from form to meaning components, and a metaphorical mapping from the meaning components (the source domain of the metaphor) to the target domain of the metaphor.

As we demonstrate in this paper, our own work on metaphors in sign languages, based on Taub’s model, shows that indeed metaphors in sign languages work a bit differently from spoken languages. First, some metaphors that are very common in spoken languages cannot receive a metaphorical interpretation in the signed modality (Meir, 2010). Second, while in spoken languages a word or an expression that are interpreted metaphorically have the same form as their non-metaphorical counterpart, in sign languages often metaphorical use of sign also involves slight changes in the form of the sign (Cohen and Meir, 2014). Furthermore, in sign languages similes are often less favored than their metaphorical counterparts, in linguistic environments in which both are acceptable in spoken languages.

In the current paper we explore some of the ways in which metaphors in sign languages differ from metaphors in spoken languages, and suggest explanations to these differences. We further argue that the study of metaphor in the signed modality offers novel insights concerning the nature of metaphor in general, and the role of figurative expressions in language.

In what follows, we describe and account for three types of differences between metaphors in sign languages and in spoken languages. In Section “The Interaction Between Iconicity and Metaphor,” we focus on the interaction between iconicity and metaphor, showing that the iconicity of signs constrains the metaphorical interpretations they can get. We introduce the Double Mapping Constraint (Meir, 2010), and suggest that it can explain the differences between languages in the two modalities, as well as shed light on the predication nature of metaphor. Body-part terms, a common source for metaphors in spoken languages, show variable behavior concerning participation in metaphorical expressions in sign languages. We argue that this variable behavior can be explained by the interaction between the DMC and Croft’s (2003) constraint (see section “The Body in Metaphors”). Croft’s constraint is also used to explain two additional, seemingly unrelated, differences between sign and spoken languages, namely that in sign languages similes are often dispreferred while their metaphor counterparts are acceptable, and the fact that often metaphorical signs have a slightly different form than their non-metaphorical counterparts (see section “Similes and Metaphors in ISL”). We conclude by describing two intriguing difference between the use of metaphors in signed vs. spoken languages, to which we do not yet have an explanation, and which we leave for future research (see section “Conclusions and Future Work”).

A word on methodology is in order here. The data presented in this paper are based on consultation with three ISL native signer, and an ASL native signer, as well as some informal discussions with a few more fluent ISL signers. Though there are differences and variation among signers regarding specific possible and impossible metaphors and figurative expressions in ISL, there was general agreement regarding the data presented here.

## The Interaction Between Iconicity and Metaphor

### The Double-Mapping Constraint

Metaphor involves mapping between source and target domains. However, not any such mapping is acceptable. For example, Lakoff (1990) formulates what he calls the Invariance Hypothesis, according to which metaphorical mappings between source and target domain are partial, and the portion of the source domain which is mapped preserves the image schematic structure of the source domain that is topologically consistent with the structure of the target domain. Thus, metaphors only map structure from the source domain that is compatible with the target domain.

Meir (2010) notes that in sign languages metaphorical mapping is further constrained, as some expressions that receive a metaphorical interpretation in spoken languages cannot be so interpreted in sign languages. For example, (1–3) normally do not mean that the house/acid/car literally ate all my savings/the metal/gas, but rather that these substances were consumed by the event that took place.

(1) The house ate up all my savings.
(2) The acid ate through the metal.
(3) My car eats gas.

However, this metaphorical interpretation of the verb *to eat* is unavailable when these sentences are translated to sign languages, such as American or Israeli Sign Languages. Meir attributes the unavailability of metaphorical interpretation to the iconicity of the sign EAT in these languages, whose form represents putting something into the agent’s mouth (**Figure 1** above). She suggests that the iconicity of this sign clashes with the shifts in meaning that take place in these metaphorical extensions. This explanation is based on Taub’s (2001) model that both iconicity and metaphors are built on mappings of two domains: form and meaning in iconicity, source domain and target domain in metaphors. Iconic signs that undergo metaphoric extension are therefore subject to both mappings.

Yet, this double mapping is not always available. When the two mappings do not preserve the same structural correspondence, Meir (2010) argues that the metaphorical extension is blocked. This line of explanation accounts for the impossibility of using the ISL sign EAT in the above expressions. The meaning of ‘eat’ is ‘to put (food) in the mouth, chew if necessary, and swallow.’ That is, the food is consumed as a result of the eating event. But the consumption of the food is not represented iconically in the form of the sign. The form of the sign iconically represents holding a small object (by the 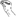 handshape), and putting it into the agent’s mouth (represented by the movement of the hand toward the signer’s mouth). Each of the formational components of the sign (its handshape, location and movement) corresponds to a specific meaning component of the event of eating, as is shown in the left and middle columns of **Table 1**.

**Table 1:** Double mapping for EAT and ‘consuming is eating.’

Iconic mapping		Metaphorical mapping
**Articulators**	**Source**	**Target**

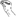 handshape Mouth Inward movement ***X***	Holding an object (food) Mouth of eater Putting food into mouth Consumption of food	***X*** ***X*** ***X*** Consumption of object

But the metaphorical use of *eat* in the above sentence profiles the consumption: *The house ate up my savings* means that the house consumed my savings as the agent consumes the food in an eating event. The metaphorical mapping between the two domains is presented by the middle and right columns of **Table 1**. The two mappings, the iconic mapping and the metaphoric mapping, do not match, as can be seen from **Table 1** (Meir, 2010, p. 879).

The meaning component that is active in the metaphorical mapping, the consumption, is not encoded by the iconic form of the sign. And the meaning components of the iconic mapping – the mouth, manipulating an object, putting into mouth – are bleached in the metaphor. The mismatch in the double mappings of the verb EAT and its intended metaphorical interpretation suggests that there is some kind of interaction between the iconic form of a sign and the kinds of metaphorical extensions it can undergo. Specifically, the iconic form of a concept and its metaphorical extension cannot profile different aspects of that concept. This is captured in the following constraint (Meir, 2010, p. 879):

The Double-Mapping Constraint (DMC): A metaphorical mapping of an iconic form should preserve the structural correspondences of the iconic mapping. Double-mapping should be structure-preserving.

**FIGURE 3:**
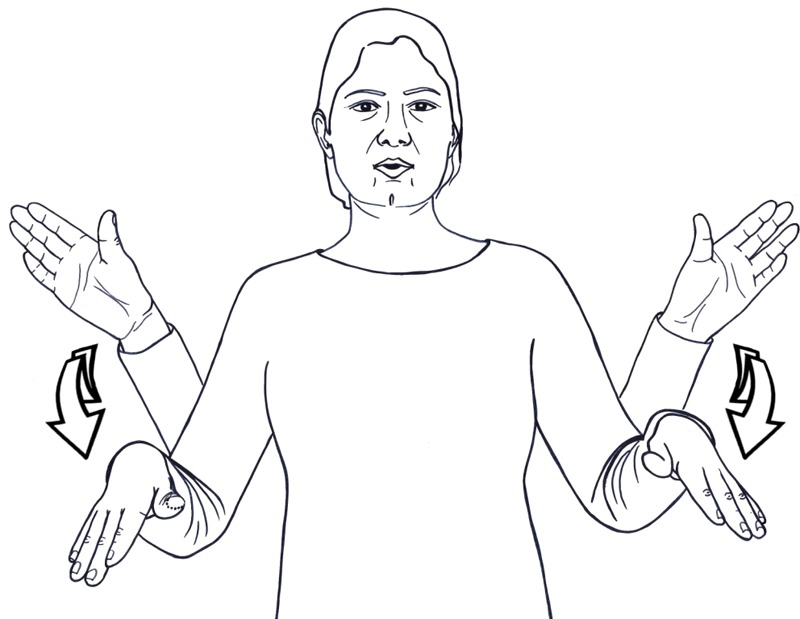
The iconic ISL sign FLY, highlighting the manner of motion.

The DMC can account for other metaphors that are possible in many spoken languages but not in sign languages, such as *Time flies, He climbed the ladder of success, the project took off*. In each of these expressions, the concept undergoing metaphorical extension is represented in ISL and ASL by an iconic sign, whose form highlights aspects of the meaning that should be bleached in the metaphor. In FLY (**Figure 3**), the hands represent the flapping of the wings, a meaning component irrelevant for the metaphor. The metaphor profiles the speed of motion, which is not represented by the form of the sign. Similarly, the form of the ISL sign CLIMB highlights the manner of motion (moving by grasping the wrings of the ladder in an alternating fashion) rather than the upward movement intended as the basis for the metaphoric interpretation; and the form of the ISL sign TAKE-OFF highlights (by its 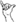 handshape) the instrument performing the action (an airplane), which is irrelevant for the metaphor.

### The Source for the DMC: Inhibition in Metaphor and Iconicity

The DMC suggests that iconicity interacts with metaphor in an interesting way: it restricts the possibility of an iconic sign to be used or interpreted metaphorically, if the property which is iconically represented in the form of the sign is not the property which the metaphor is based on. But what is the source for this restriction? Why does iconicity interfere with metaphorical extension of a sign? We attribute this interference to another property of iconic expressions, the fact that iconicity cannot be inhibited. Yet metaphorical interpretation requires the inhibition of certain properties of the word. It is the tension between these two factors that will feature prominently in our explanation of the DMC. Inhibition, then, is crucial to our suggestion, to which we turn in this section. We first look at the role of inhibition in metaphoric interpretation, and then at its interaction with iconicity.

#### Metaphor and Inhibition

Intuitively, in order to interpret a metaphoric statement such as (4), we need to inhibit the literal properties of the sun, such as being very massive or very hot or 150 million kilometers from Earth; we keep only the properties that are relevant to the interpretation of the metaphor. The notion that metaphor interpretation requires inhibition has received some experimental confirmation.

Glucksberg et al. (2001) show that properties that are not relevant to the metaphorical interpretation are negatively primed, i.e., inhibited. Subjects were asked to judge the acceptability of target sentences following either metaphors or literal statements. For example, a sentence like (6) was judged following either the literal (5a) or the metaphorical (5b).

(4) Juliette is the sun.
(5) (a) The hammerhead is a shark.
  (b) My lawyer is a shark.
(6) Geese can swim.

Glucksberg et al. (2001) found that (6) took longer to judge when preceded by (5b) than when preceded by (5a). Their explanation is that the word *shark* normally primes the property *swim*, facilitating the interpretation of a sentence containing it. However, if *shark* is interpreted metaphorically, this property is not primed. This is why (6), which contains the word *swim*, takes longer to judge when following the metaphorical (5b) than when following the literal (5a).

Fernandez (2007) extended these findings, by showing that the irrelevant property is not simply not primed, but actually inhibited. Using a lexical decision task, Fernandez showed that words that are related to the literal meaning of the metaphor actually took longer to judge than words that were not related at all.

For example, consider (7) and (8): in both of them, a target word follows a sentence. The target word in (7b), *skin*, is not related to any of the words of (7), hence it is not primed. In contrast, the target word in (8b), *animal*, is related to the word *zoo* appearing in (8a). But note that *zoo* is used metaphorically in (8a), and the interesting result is that the judgment of (8b) is actually *slower* than the judgment of (7b)! This result demonstrates that the literal meaning of *zoo* is actually actively inhibited, not merely not primed. Interestingly, the effect occurs only after 1500 ms, which is consistent with the fact that inhibition takes time.

(7) (a) Wisdom teeth are troublemakers.
  (b) Skin
(8) (a) State schools are zoos.
  (b) Animal

Langdon et al. (2002) provide evidence for the inhibition hypothesis from a different direction: the behavior of schizophrenic patients. In particular, they studied both the ability of these patients to inhibit irrelevant information and their interpretation of metaphors, and found the following correlation: “the better the patients were at suppressing prepotent inappropriate information… the more likely they were to recognize appropriate uses of metaphorical speech.”

#### Iconicity and Inhibition

**FIGURE 4:**
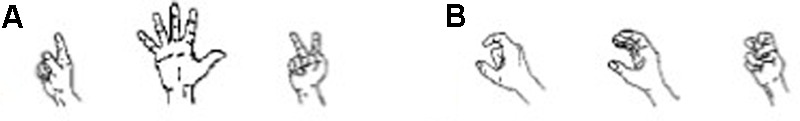
Examples of handshapes with **(A)** straight fingers and **(B)** with curved fingers.

Thompson et al. (2010) found that iconic signs are much harder to inhibit than non-iconic ones, even in tasks that require no access to meaning. They asked deaf signers of British Sign Language (BSL) to make a phonological decision: to decide whether BSL signs, presented in video clips, were produced with a handshape with straight or curved fingers (see **Figure 4**). The signs were both iconic and non-iconic, but importantly, the iconicity of the signs was irrelevant for the task, as the task did not involve access to the meaning or meaning components of the signs. Thompson et al. (2010) found that iconic signs led to slower reaction times and more errors in the participants’ responses. They suggest that meaning is activated automatically for highly iconic signs, because of the closer form-meaning mapping in these signs^2^. This automatic activation of meaning interfered with the task because it provided information that could not be inhibited yet was irrelevant to the task at hand. It seems, then, that iconicity cannot be ignored, even when it is irrelevant.

Another possible inhibitory effect of iconicity was found by Baus et al. (2013). The tasks in this study did involve meaning, as bilingual (ASL-English) signers were asked to translate signs (iconic and non-iconic) from ASL to English and from English to ASL, or to determine whether a given ASL sign and a given English word match in meaning. The findings show that iconicity interfered with the performance of fluent ASL-English bilinguals: their responses to the ASL-into-English translation task and the matching task were significantly slower for iconic signs than for non-iconic ones. These results are surprising. In the Thompson et al.’s (2010) study described above, iconicity seemed to interfere with the task because it caused automatic access to meaning, which was irrelevant to the phonological task in that study. Yet in the translation task, faster access to meaning is expected to speed translation for iconic signs. The authors suggest that maybe the iconicity of the signs “forced” the participants to use a specific translation strategy that slowed down performance. In order to translate a word, an association must be formed between the lexical systems of the source and target languages (*word–word association*), or the associations can be formed through the conceptual systems (*conceptually mediated translation*). The authors tentatively suggest that “the imagistic or sensory-motor properties of the iconic signs induced these signs to be translated via conceptual mediation, which slowed translation times” (Baus et al., 2013, p. 269). An explanation along these lines supports the hypothesis that iconic properties of signs cannot be inhibited.

#### Putting it Together: The Source for the DMC

It seems, then, that iconicity and metaphorical interpretation play a constant tug-of-war game. Metaphorical interpretation requires the inhibition of some aspects of the literal meaning of the word, in particular, those aspects that are irrelevant for the metaphorical reading. Iconic aspects of signs, together with the meaning components they are associated with, on the other hand, cannot be inhibited. They are too salient in the form of the sign to ignore. If the metaphorical reading requires the inhibition of those meaning components that are iconically present in the form of the sign, the metaphoric interpretation is not available. Hence the source for the DMC is the competing and opposing forces that iconicity and metaphor require: inhibition of meanings vs. the impossibility of inhibition of these meanings. ^3^We now turn to a specific type of source domain for metaphor that is affected by the DMC in an interesting way, namely body-part terms.

## The Body in Metaphors

### The Problem

**FIGURE 5:**
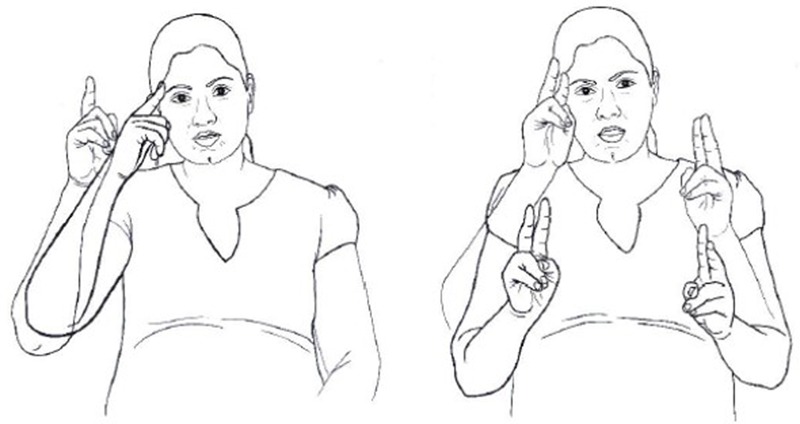
Compound-like constructions that include body part in ISL: HEAD+STOP ‘to have a blackout.’

Words denoting body-parts are a rich source for metaphorical use in spoken languages, especially for expressing spatial and containment relations (‘the foot of the hill’), part-whole relations (‘the mouth of the river’), and more abstract relations (‘the heart of the problem’). In ISL and other sign languages, such metaphors are completely absent. They are also impossible; signers we’ve consulted with affirm that that they would never use body-part signs in such contexts. Yet sign languages use body-part signs productively in compound-like constructions, such as the following ISL examples: HEAD+STOP ‘to have a blackout’ (**Figure 5**), HEAD+FALL ‘to faint,’ HEAD+COGWHEELS ‘to think deeply,’ ‘EYE+SHARP’ ‘to discern visually,’ MOUTH+SMEAR ‘to mislead (by talking),’ HEAD+EMPTY ‘doesn’t understand anything.’ These constructions are common in various sign languages, such as ISL, ASL, British Sign Language (BSL) and others^4^. In ISL we found about 70 constructions of this type (termed *sense compounds* in Aronoff et al., 2005). They are also productive; signers use body-part terms with other words to create novel expressions. The equivalent English constructions are predicate-argument constructions (e.g., *My head is empty, His eyes are sharp*) or predicating-modifier constructions (an *empty-headed person, sharp-eyed*).

Why do sign languages allow metaphors involving body-part signs in these constructions but not in relational constructions, while spoken languages allow both? What is special about body-part terms in sign languages that makes them more constrained in terms of the metaphorical extensions they can undergo? The answer to this puzzle involves both the DMC, and a constraint suggested by Croft (2003) regarding the autonomy and dependency of elements in a metaphorical construction. We turn now to introduce Croft’s constraint, then return to offer an explanation of the behavior of body-part signs in metaphors in sign languages.

### Croft’s (2003) Constraint

Croft (2003) addresses the issue of what drives listeners to interpret a construction metaphorically rather than literally. For example, in a sentence such as *Denmark shot down the Maastricht treaty* (ibid., 162), how does the listener know that the sentence is about politics rather than about war? *Denmark* and *the Maastricht treaty* are entities in the domain of politics,^5^ while *shoot down* is an action that belongs to the domain of war. Why is *shoot down* interpreted as a political action rather than interpreting *Denmark* and *the Maastricht treaty* as belonging to war? And why not interpret the sentence literally? Croft argues that what drives the metaphorical interpretation is “the conceptual unity of domain: all of the elements of a syntactic unit must be interpreted in a single domain.” (ibid., 162). If the literal interpretation provides different semantic domains, the sentence is not rejected as semantically incoherent. Rather, the listener attempts to interpret some of the elements figuratively, as belonging to the same semantic domain as the other elements in that sentence (ibid., 195).

Yet which element of the unit will be interpreted metaphorically? Here Croft draws on Langacker (1987, 1989, 1991, 2002) distinction between autonomous and dependent elements. Langacker notices that in most grammatical combinations, one notion is relatively autonomous, while the other is relatively dependent in the sense that it presupposes the autonomous element as part of its internal structure or interpretation (Langacker, 2002, p. 122). In the phrase *a tall man, man* is autonomous, since one can conceive of a man without considering his height; while *tall* is dependent, since its meaning is dependent on the conceptualization of an entity to which a quality of tallness can be attributed (Sullivan, 2009, p. 3). When considering predicative elements such as verbs, adjectives, or adverbs vs. nominal arguments, it is usually the case that the latter are autonomous while the former are dependent.

Croft suggests that this distinction is relevant for figurative interpretation of language. In particular, in metaphor, Croft observes that the dependent element is interpreted metaphorically, while the autonomous elements are interpreted non-metaphorically, and signal the target domain. In the sentence above, *Denmark* and *the Maastricht treaty* are autonomous, while *shoot down* is dependent, as its meaning is elaborated by the two nominal phrases. Therefore, the two nominal phrases are interpreted non-metaphorically, and they indicate the target domain (politics) onto which the dependent element should be mapped. The verb, the dependent element, receives a metaphorical interpretation: its meaning is mapped from the domain of war (its source, or literal domain) to the domain of politics (the target domain).

To take another example, in the sentence *My heart broke*, the noun *heart* and the verb *broke* belong to two semantic domains: *heart* belongs to the domain of emotions (by metonymy, the heart is the location of emotions) while *break* belongs to the domain of solid objects. Domain unity requires both elements to be interpreted as belonging to the same domain. Since *break* is dependent while *heart* is autonomous, it is *break* that is interpreted metaphorically. *Heart* signals the target domain of emotion, while *break*, whose source domain is that of solid objects, is mapped to the domain of emotions, receiving a metaphorical interpretation.

Evidence for this constraint comes from psychological experiments. Gentner and France (1988) found that subjects prefer to generate metaphorical interpretations for verbs rather than nouns. For example, they prefer an interpretation of (9) where the lizard basked in the sun, rather than an interpretation where the person who looks like a lizard worshipped.

(9) The lizard worshipped.

There is also evidence from corpus studies. Huang (1994) found that in both English and Chinese, metaphorically interpreted elements are predominantly adjectives and verbs, not nouns. Sullivan (2007, 2009), in a corpus study of metaphorical expressions, tested Croft’s constraint. She analyzed 2415 metaphorical constructions of six different types. Her findings indicate that Croft’s predictions are borne out in each of these six constructions. Two of these constructions belong to the constructions relevant for the sign language data, presented above, to which we turn in the next section.

What is the explanation for Croft’s constraint? Croft does not propose one, but we believe it follows from the nature of metaphor. There is a debate concerning what the interpretation of metaphor involves^6^. The prevailing views can be roughly classified into two camps: Class inclusion and Predication.

Consider the following example:

(10) Businesses are dictatorships.

According to class inclusion theories of metaphor, the interpretation of (10) involves the construction of an *ad hoc* superordinate class—dictatorship*. This class plausibly contains organizations and communities that are managed non-consensually and punitively by one person. Sentence (10) is then taken to mean that the class *business* is a member of this *ad-hoc* superordinate class.

According to predication theories of metaphor, the interpretation of (10) is different. It assumes that there is a set of relevant properties associated with dictatorships: be a form of government, be ruled by one person, be ruled non-consensually, regulate many aspects of the lives of their members, employ political propaganda, use terror and violence, etc. One such property, *P*, is selected. Sentence (10) then means that business have property *P*. For example, in a given context the selected property may be *be ruled by one person*. Then (10) means that businesses are run by a single person. In this paper we assume the predication theory of metaphor (cf. Cohen and Meir, 2015). One argument for the predication view is that it makes possible a natural explanation of Croft’s constraint, which would otherwise be an unmotivated stipulation. The explanation is as follows. An intuition that goes as far back as Plato and Aristotle is that a sentence is divided into subject and predicate, where the subject is typically nominal and the predicate is typically verbal or adjectival^7^. It follows that the prototypical predicative categories are verbs and adjectives, rather than nouns (although all three have the same logical type: properties of individuals). We therefore expect verbs and adjectives to be preferred in metaphorical interpretation, which is precisely what is described by Croft’s constraint.

### Possible and Impossible Body-Part Metaphorical Extensions in Sign Languages

We turn now to the participation of body-part terms in metaphors in sign languages. Let us address first the question of why body-part terms in sign languages are more constrained than their spoken language equivalents. Again, the key to that question is their form. Body-part terms in sign languages usually take the form of pointing to the relevant body part. The signs for EYE, NOSE, EAR in ISL involve a pointing handshape (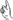) to the relevant organs. The sign for HEAD is a 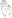 handshape that touches the temple; FACE involves a circle movement of the 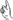 handshape around the face; HEART is a 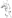 hand that touches the location of the heart, and so on. In all these signs, the actual body part serves as the place of articulation of the sign, and is highlighted by the movement of the hand toward it.

The salience of the actual body part in the sign is also what constrains its use in metaphors. The foot of the hill is not really a foot; it is the lowest part of the body, the one that makes contact with the ground, which is what it has in common with the feet of a human body. But it doesn’t have toes, it is not connected to a leg, and it doesn’t come in pairs. In spoken languages, the metaphorical use is built on the resemblance of the spatial relations between the foot and the body it is part of, abstracting away from the actual form of the human vs. geographical foot. In sign languages, the actual form of the organ is there as part of the form of the sign, and is highlighted in the sign. It cannot be inhibited, and its actual form cannot be ignored. The metaphorical mapping is therefore incongruent with the iconicity of the sign, violating the DMC, and is consequently blocked.

Yet body-part terms in sign languages seem to be absent from one type of construction, and possible in another type. This differential behavior can be explained by looking at the relationship between the different components of each construction in terms of their relative dependency. In constructions such as *the mouth of the river, the foot of the hill* (Sullivan’s ‘prepositional phrase constructions’), there is a part-whole relationship between the body-part and the noun in the PP, designating a geographical area. The element denoting the part is the dependent element, since its conception is dependent on the conceptualization of the whole that it is part of. It is impossible to conceive of a mouth without referring to body that it is part of (in our case, river). The entity is relatively autonomous, as it is possible to conceive of a river (or of any body) without referring to specific sub-parts of it (Croft, 2003). According to Croft’s constraint, the NP denoting the entity, as the autonomous element, is interpreted literally and signals the target domain (geographical areas), while the body-part, as the dependent element, should receive a metaphorical interpretation. However, in sign languages this is not possible, as pointed out above: the form of signs denoting body-parts highlights the actual body-part. The metaphorical mapping is therefore incongruent with the iconicity of the sign, violating the DMC, and is consequently blocked.

The metaphorical expressions HEAD+EMPTY, HEART+BLACK, exhibit a different pattern of autonomous-dependent relationship between its components. These belong to what Sullivan (2007, 2009) calls ‘predicating modifier constructions’ (*an empty head*), or to ‘predicate-argument constructions’ (*your head is empty*). In both cases, the predicating element is conceptually dependent, since its interpretation needs to make reference to an entity to which the relevant property can be attributed. The body-part is autonomous, signaling, by metonymy, the target domain of the metaphor: mental activities or emotions (the head is the site for mental activities, the heart the site of emotions). Since the body-part does not receive metaphorical interpretation, it is not subject to the DMC, and is not blocked by it. Therefore, such constructions are possible in sign languages.

We conclude that body-part signs are indeed excluded from being used metaphorically in sign languages because of their form. But they can be part of a metaphorical construction where they function as the autonomous element, denoting the target domain. The interaction of the DMC with Croft’s constraint explains how body-part signs, and iconic signs in general, can participate in metaphors.

## Similes and Metaphors in ISL

### The Distribution of Similes vs. Metaphors in ISL

The phenomena described in the previous sections indicate that metaphorical use is more constrained in sign languages than in spoken languages, which we attributed to the constraining effect of iconicity on metaphor. Since iconicity is much more prevalent in sign languages, this restricting effect is more noticeable in these languages than in spoken languages.

We now turn to another phenomenon where ISL exhibits a more restricted use of figurative language compared to English and Hebrew: the use of similes. Similes are figures of speech that involve comparison between two things of different kinds, in order to characterize one term by the other. In that, they resemble metaphors. However, in similes, the comparison is made explicit, by using words such as *like, as*: *My lawyer is like a shark, He works like a mule*. Importantly, in many linguistic structures in spoken languages, similes and metaphors can be both used, as in (11):

(11) John is (like) a snake.

The relationship between similes and metaphors has been studied extensively for millennia. Starting with Aristotle, many scholars (e.g., Bergmann, 1979; Miller, 1993; van Genabith, 2001) argue that a metaphor is an (elliptical) simile. Yet, others argue that metaphors and similes differ in kind. How do we decide this question? We may try to find languages where similes are allowed but not metaphors, or vice versa. It would seem that if such a language is attested, this would indicate that metaphors cannot be reduced to similes. It turns out that the study of sign languages provides us with such a language, but not with the expected outcome.

To the best of our knowledge, the use of similes vs. metaphors has not been studied in sign languages. It might be expected that since in similes the comparison is explicit, iconicity will not play such a restrictive role regarding their use. On the other hand, since in both metaphors and similes, the characteristics that are profiled by the comparison do not necessarily coincide with those profiled by the iconic form of a sign, similes may show very similar behavior and distribution to that of metaphors. To our surprise, when we started looking at the distribution of metaphors and similes in ISL, we found out that in some environments, such as predicative or adverbial positions, similes are often not possible or dispreferred, where metaphors are possible or preferred, as exemplified in (12–14):

(12) JOHN (^∗^LIKE) SNAKE ‘John is (^∗^like) a snake.’
(13) MARY WORK (?LIKE) MULE ‘Mary works (?like) a mule.’
(14) KIM STRONG (?LIKE) OX ‘Kim is strong (?as) an ox.’

Note that in the English sentences that correspond to (13) and (14), not only is the simile form possible, but it is, in fact, mandatory: the metaphor form, that is, the form without *like/as*, is unacceptable.

What is the source of these differences between the two types of languages? To answer this question, let us again consider the English sentence (11). In this sentence, under either its metaphor or simile form, the noun *snake* receives a figurative interpretation. But doesn’t this fact violate Croft’s constraint? Nouns are regarded as relatively autonomous, and should not receive figurative interpretation according to Croft. However, Croft (2003) speculates that the noun can be construed as dependent after all: “While there appears to be no general principle by means of which we can say that the metaphorically interpreted noun is… dependent… it seems to be a not unreasonable hypothesis… and should be investigated further” (p. 194). But sign languages allow another option, namely a shift in the lexical category of the noun.

### Categorical Reinterpretation of Figurative Signs

Sign languages in general show more flexibility regarding lexical categorical distinctions, in that words in many sign languages are often multicategorial and can be interpreted as nouns, verbs or adjectives (Meir, 2012 and references therein).

“We also find a substantial amount of systematic ambiguity or vagueness in many sign languages. For instance, in Indo-Pakistani Sign Language (IPSL) many signs tend to have rather general meanings that are narrowed down by the context of the utterance…. and similar problems are encountered in many other sign languages as well.” (Schwager and Zeshan, 2008, p. 513).

In ISL, for example, a sign such as LONELY may function as an adjective in (15) and as a noun in (16), and this is characteristics of many signs. In many cases, lexical category is assigned according to the function of a sign in a specific syntactic environment rather than as a lexical property of that sign.

(15) I LONELY ‘I am lonely.’
(16) LONELY MORE MORE WIDE-SPREAD ‘Loneliness becomes more and more wide-spread.’

We propose that in the case of nominal metaphor, the noun is reinterpreted as an adjective or adverb, and then it is more readily be construed as dependent. For example, in (12’) *SNAKE* is interpreted as an adjective:

(12’) HE SNAKE ‘He (is) snaky/snakelike.’

**FIGURE 6:**
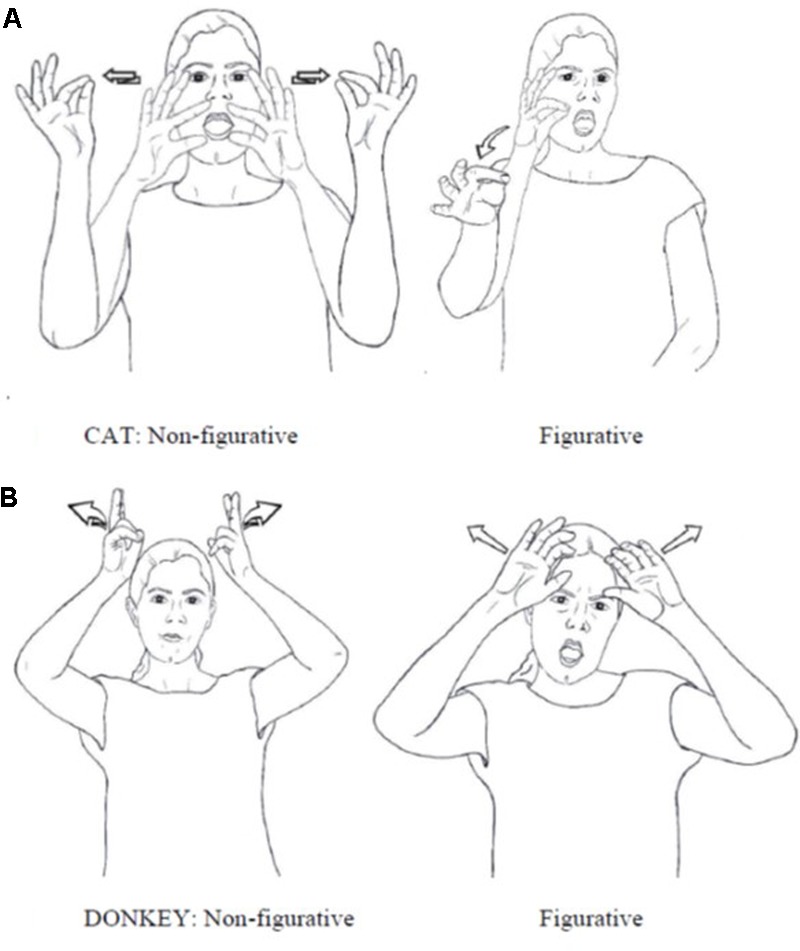
Differences in form between figurative and non-figurative use of ISL signs: **(A)** CAT. **(B)** DONKEY.

One piece of evidence for categorical reinterpretation of figurative signs comes from the fact that many metaphorical signs in ISL and ASL have a slightly different form from their non-metaphorical counterparts. This has been observed for ASL by Klima and Bellugi (1979, p. 299) “Figurative extensions of meaning are preferentially accompanied by minimal changes in movement.” In ISL, we find that the difference in the quality of movement (as in the sign CAT, **Figure 6A**) is often accompanied by other phonological differences such as the number of hands (non-figurative CAT is two-handed while figurative CAT is one-handed) and handshape (as in DONKEY, **Figure 6B**). However, the quality of movement is crucial here, since it is often associated with differentiating parts of speech in sign languages. For example, nouns and verbs in noun-verb pairs in many sign languages are distinguished by length and quality of movement. This observation was first made for ASL by Supalla and Newport (1978), and then found in other sign languages (see Schwager and Zeshan, 2008; Tkachman and Sandler, 2013 for an overview). Furthermore, ASL has means for deriving verbal/adjectival predicates from nouns. Klima and Bellugi (1979, p. 296) describe a systematic change to the movement of ASL nouns, forming predicates with the meaning of ‘to act/appear like X,’ as in ‘to act like a baby’ from BABY, ‘to seem Chinese’ from CHINESE and ‘pious’ from CHURCH. The derived predicates have a fast and tense movement with restrained onset. Similarly, differences in movement encode an extended use of signs as sentential adverbials, as in ‘suddenly’ or ‘unexpectedly’ from WRONG, ‘unfortunately’ from TROUBLE.a

A second piece of evidence demonstrating that nominal metaphors are indeed interpreted as adjectives is the fact that they can be modified by a degree adverb such as ‘very’ (17a). Such modification is also possible with regular adjectives (17b), but impossible when the noun is used literally (17c).

(17) (a) HE CAT_figurative_ VERY. ‘He is very “catty”/sly.’
  (b) HE STRONG VERY. ‘He is very strong.’
  (c) ^∗^HE CAT_literal_ VERY. ‘He is very cat.’

### Back to Similes vs. Metaphors

The proposal that metaphorically interpreted-nouns are reinterpreted as adjectives explains why similes are strongly dispreferred in ISL: syntactically, the preposition LIKE cannot precede an adjective or an adverb.

(18) JOHN (^∗^LIKE) SNAKE_figurative_

This is to be contrasted with a spoken language like English, where the figuratively interpreted noun does not change its category, and can therefore unproblematically combine with a preposition:

(11’) John is (like) a snake.

Note that this explanation is essentially syntactic, and is not dependent on any difference in meaning between metaphors and similes. Even if metaphors are, indeed, elided similes, the scarcity of similes in sign languages would still be explained in the same way, on the basis of the categorial flexibility of these languages.

There is an important lesson here. In an attempt to demonstrate that metaphors and similes differ in their meaning, we tried to find languages that have metaphors but (almost) no similes. We have, indeed, found such a language—ISL; and yet, we found that the facts of this language have nothing to do with any supposed semantic difference between metaphors and similes. Hence, if anything, our findings provide support for the view that metaphors and similes *are* very close in their meaning.

## Conclusion and Future Work

In the preceding sections, we focused on the differences between manual-visual languages and auditory languages in the expression and use of metaphor and simile. A key issue here is the greater ability of sign languages for iconic expressions. While iconicity provides signs with the ability to represent visual aspects of concepts in a vivid and straightforward way, it also constrains those signs from taking additional, metaphorical meanings, if these are not built on the visual imagery profiled by the sign’s iconicity. As we pointed out, iconicity cannot be inhibited, while metaphorical interpretation is built on inhibition. If both forces play tug-of-war on the same meaning components, the metaphorical interpretation is blocked. The use of similes is further constrained by the categorical shift from nouns to adjectives/adverbs. This shift is made possible by the general flexibility of sign languages regarding lexical categories. But it also constrains the use of similes, since a preposition such as LIKE must be followed by a noun, not an adjective/verb.

We would like to conclude by describing two striking difference between the use of metaphors in signed vs. spoken languages, for which we do not yet have an explanation.

### The Expression of Metaphor

We have seen in Section “Categorical Reinterpretation of Figurative Signs” that when a sign is interpreted metaphorically, its form changes slightly. This constitutes an interesting difference between languages in the two modalities: in spoken languages, the main expression of metaphor is through the use of a word in a different semantic domain with an accompanying change in meaning, as in *wave* (*an electro-magnetic wave, waves of immigrants, feminism wave, wave of excitement*); in sign languages, in contrast, the main expression of metaphor is in creating new signs (Taub, 2001; Roush, 2016). Sign language abound with metaphorical signs, signs built on both iconic and metaphorical mapping, such as the sign LEARN (**Figure 2**). Crucially, it is not the case that the sign EAT itself is used metaphorically; rather, the form of the sign is changed in a specific manner—movement toward the signer’s temple rather the mouth—and a *new* sign is formed.

In fact, many (if not most) of the signs denoting abstract concepts in a given sign language are built on this double mapping, as illustrated and exemplified in depth by Taub (2001). Moreover, this is a very productive way for creating new signs, in everyday use and in sign language poetry. In spoken languages, metaphor is often described as a process of making novel use of existing means: existing lexical items are used to refer to novel concepts by means of metaphorical extensions. In sign languages, this description is not accurate: metaphor is usually not making novel use of existing means, but rather the means for creating novel forms. At present, it is not clear to us how to account for this difference, and we leave it as an open question for future research.

### Alternatives to Metaphor

Another difference between languages of the two modalities pertains to providing alternatives to metaphors. Metaphors and similes are often used to create a vivid sensory image. But there are other means for achieving this goal. One alternative way to create vivid imaginary is through iconic means, which, as we have seen above, cannot be inhibited.

**FIGURE 7:**
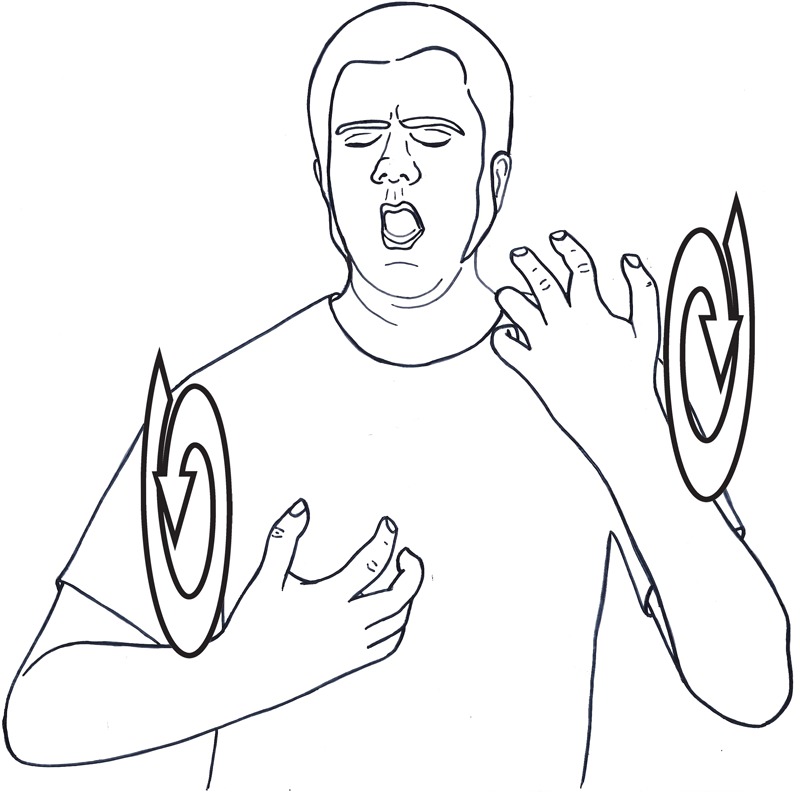
Body parts signal target domain of metaphor: VERY-ANGRY (boiling signed close to chest, signaling the domain of emotions).

**FIGURE 8:**
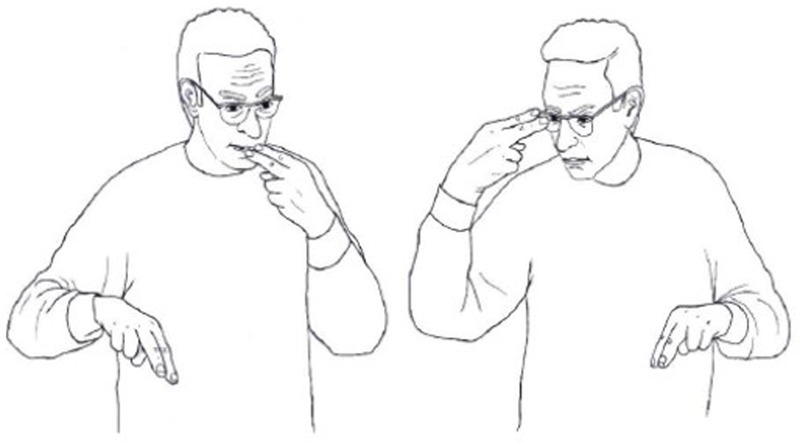
Body parts signal target domain of metaphor in sign language poetry: the member of parliament consumes food and news, by his mouth and eyes (Wim Emerik, ‘Member of Parliament’).

Since iconicity is much more prevalent in sign languages, we expect to find many instances of vivid iconic representations of the desired visual image, instead of metaphors or similes. This is indeed the case. For example, while the salience of body-parts can inhibit certain metaphorical uses, as we discussed in Section “The Body in Metaphors,” it can also be exploited for specific effects, both in everyday use and in poetic signing. A widespread use of body parts in visual languages is signaling the target domain of metaphorical mapping by articulating a sign close to a specific body part. We saw an example with the sign LEARN, where the head signals that the action encoded in the sign is a mental action. Another example is the sign for BOIL, usually articulated in neutral space. However, when signed close to the chest, the metaphorical site for emotions, it means ‘inner boiling,’ that is, VERY-ANGRY (**Figure 7**). Changing the sign’s location can be also used creatively. One of our consultants signed the sign DEPLETE on his bicep instead of in neutral space to convey the meaning of ‘to be exhausted, run out of steam’ (Meir and Sandler, 2008, p. 57). Another consultant created a new sign by signing the sign SHINE close to the eyes, to convey the meaning ‘shining eyes.’ The consulted pointed out that this neologism is more vivid in evoking a mental image of shining eyes than using a metaphor such as ‘*Her eyes were shining stars.*’ The deaf Dutch poet Wim Emerik, in his poem ‘Member of Parliament,’ uses the same technique to convey the idea that the politician consumes the reported news as an automatized bodily function. As the poet depicts the politician eating lunch and reading the newspaper, he changes the location of the verb EAT from the mouth to the eyes, indicating that the politician consumes news as he consumes food (**Figure 8**) (ibid., 56)^8^.

All these examples show how the iconicity of body parts in sign languages can be exploited to create a vivid sensory image, without resorting to explicit or implicit comparisons as in similes and metaphors. Spoken languages cannot exploit body-part terms in the same way, since these terms are not iconic in the spoken modality. Yet iconicity can be used to create sensory image even in the spoken modality. Languages that have a wide array of mimetics, often prefer those to the use of metaphors: “… some types of pain that are mimicked by mimetics in Japanese are expressed by metaphors in other languages (e.g., *gangan* ‘one’s head pounding,’ *kirikiri* ‘one’s stomach splitting,’ *sikusiku* ‘one’s stomach griping’).” (Akita, 2016, p. 155). And Sharlin (2009, p. 5) concludes: “Ideophonics, flexible and welcoming to creativity, seem to take the place of other figurative language (simile, metaphor) generally absent in Japanese.” It seems, then, that creating a vivid sensory image is cherished by language users in general. But different languages have different means for achieving this goal. Affordances of the modality may channel languages to use specific means, e.g., the preference of languages in the signing modality to use iconic expressions. But as the use of mimetics show, it is not only about modality. Even within the same modality, languages may show different preferences. We leave it for future study to investigate the different factors that may lead languages to show preference to one figurative means over another.

### A Final Word

In languages in both modalities, metaphor is prevalent, and many metaphorical mappings are shared by both, providing strong support for a universalist view of metaphor and metaphorical mapping in language. But each modality has its own constraints and affordances, shaping the use of metaphors in ways particular to the modality. A cross-modal and cross-linguistic comparison enables us to grasp the central role of metaphors in human expression on the one hand, and the different means that languages provide for carrying out this function.

## Author Contributions

All authors listed have made a substantial, direct and intellectual contribution to the work, and approved it for publication.

IM had tragically passed away before the paper was published. She was a brilliant linguist and a wonderful colleague, and will be sorely missed by all who knew her.

## Conflict of Interest Statement

The authors declare that the research was conducted in the absence of any commercial or financial relationships that could be construed as a potential conflict of interest. IM has been collaborating on research projects with host editors Wendy Sandler and Carol Padden over the past two decades.

## Footnotes

1 Forceville (1996, 2005) draws a distinction between pictorials metaphors and what he calls ‘similes,’ but by this he just means cases where the source and target domains are visually presented separately.

2 A similar explanation in a different theoretical framework is suggested by Emmorey (2014), who regards iconic representations as structured mapping between two mental representations. She suggests that structure-mapping sometimes cannot be avoided, and that iconic mappings are automatically available to signers.

3 This conclusion has implications, beyond sign languages, on the general meaning of metaphor. Specifically, it favors the view that metaphor expresses predication rather than categorization (Cohen and Meir, 2015).

4 See Aronoff et al. (2005); Meir and Sandler (2008) for ISL; Brennan (1990); Sutton-Spence and Woll (1999) for BSL; Carol Padden, p.c. for ASL; Meir et al. (2012) for ABSL.

5 In fact, *Denmark* is in the domain of geography, and belongs to politics only after it is interpreted metonymically. Although Croft does discuss metonymy, we will not deal with it here.

6 See Chiappe and Kennedy (2001), Glucksberg (2001, 2008, 2011), Bowdle and Gentner (2005), Glucksberg and Haught (2006a,b), Jones and Estes (2006), and Utsumi (2007, 2011), *inter alia*.

7 Of course, the subject needs not be a separate phrase or even word, and may be incorporated into the predicate, as it is in many polysynthetic languages.

8 Another creative use of the signer’s body is personification, where the poet uses his/her body to ‘become’ the entity (person, animal, object) in the poem. This creates a blend of the characteristics of the poet and characteristics of the focal entity, highlighting how those entities would view the world not only as humans but as signing deaf people, who view the world visually and communicate in signs (Sutton-Space, 2012, p. 1007. See Sutton-Spence and Napoli, 2010 for a thorough description of this device).
